# Comparison of endogenous amino acid losses in broilers when offered nitrogen-free diets with differing ratios of dextrose to corn starch

**DOI:** 10.1038/s41598-022-09746-0

**Published:** 2022-04-05

**Authors:** Huajin Zhou, Wei Wu, Tahir Mahmood, Yanhong Chen, Yanwei Xu, Youli Wang, Jianmin Yuan

**Affiliations:** 1 State Key Laboratory of Animal Nutrition, College of Animal Science and Technology, China Agricultural University, Beijing, 100193 China; 2 Adisseo Animal Nutrition, DMCC, Dubai, United Arab Emirates

**Keywords:** Zoology, Gastrointestinal system

## Abstract

The nitrogen-free diet (NFD) method is widely used to determine the ileal endogenous amino acids (IEAAs) losses in broiler chickens. Starch and dextrose are the main components of NFD, but the effects of their proportion in the NFD on the IEAAs and the digestive physiology of broilers are still unclear. This preliminary study aims to explore the best proportion of glucose and corn starch in NFD to simulate the normal intestinal physiology of broilers, which helps to improve the accuracy of IEAAs determination. For this purpose, 28-day-old broiler chickens were allocated to five treatment groups for a 3-day trial, including a control group and four NFD groups. The ratios of dextrose to corn starch (D/CS) in the four NFD were 1.00, 0.60, 0.33, and 0.14, respectively. Results noted that NFD significantly reduced serum IGF-1, albumin, and uric acid levels compared with the control (*P* < 0.05), except there was no difference between group D/CS 0.33 and the control for IGF-1. The increased Asp, Thr, Ser, Glu, Gly, Ala, Val, Ile, Leu, His, Tyr, Arg, and Pro contents of IEAAs were detected in broilers fed the NFD with a higher ratio of D/CS (1.00 and 0.60) compared to the lower ratio of D/CS (0.33 and 0.14). Moreover, ileal digestibility of dry matter and activity of digestive enzymes increased as the D/CS elevated (*P* < 0.001). Further investigation revealed that the number of ileal goblet cells and *Mucin-2* expression were higher in the group with D/CS at 1.00 when compared with group D/CS 0.33 and the control (*P* < 0.05). Microbiota analysis showed that NFD reshaped the gut microbiota, characterized by decreased microbial diversity and lower abundance of *Bacteroidetes*, and increased *Proteobacteria* (*P* < 0.05). Our results indicate that a higher D/CS ratio (1.00 and 0.60) in NFD increases IEAAs by promoting digestive enzymes and mucin secretion. However, the excessive proportion of starch (D/CS = 0.14) in NFD was unsuitable for the chicken to digest. The chickens fed with NFD with the D/CS ratio at 0.33 were closer to the normal digestive physiological state. Thus, the ratio of D/CS in NFD at 0.33 is more appropriate to detect IEAAs of broiler chickens.

## Introduction

Determination of amino acid (AA) digestibility of raw materials is the critical foundation of feed formulation in poultry production, contributing to better protein utilization and minimizing nitrogen losses. The apparent ileal amino acid digestibility (AID) is measured based on the net disappearance of ingested dietary amino acid from the proximal digestive tract to the distal ileum^1^. Dietary formulas based on the AID index have been widely employed in diet formulation. Nevertheless, AID underestimates the actual digestibility of AA in broilers by neglecting the ileal endogenous amino acids (IEAAs) losses, especially for low crude protein (CP) ingredients, such as cereal grains^2^. The IEAAs are considered an inevitable loss, consisting of salivary and gastric secretions, pancreatic and bile secretions, small intestinal secretions, mucus, sloughed epithelial cells, and microbial protein^3,4^. The formula based on standardized ileal digestibility (SID) can correct the AID by accounting for IEAAs losses induced by the protein-free ingredients. Undoubtedly, SID reflect the digestibility of feed protein more accurately, thereby measuring IEAAs losses to determine the SID of AA is necessary for accurate diet formulation.

Nitrogen-free diets (NFD) are traditionally used in monogastric animals to calculate SID, where the IEAAs losses are determined by measuring AA excretion in the ileal digesta^5^. Corn starch and dextrose are the main components of the NFD (approximately 80% of NFD), but the ratio of dextrose to corn starch (D/CS) varies in literature (0.21–3.79)^6–9^, which may, in turn, affect the flow of IEAAs. Kong et al.^10^ identified that total IEAAs were significantly higher when dextrose was used as a sole source of energy in NFD than corn starch (17,544 vs. 12,779 mg/kg of dry matter intake). Adedokun et al.^11^ also found that the IEAAs loss in NFD containing only glucose was significantly higher than that in NFD containing only corn starch (11,080 vs. 6038 mg/kg of dry matter intake). Collectively, varied ratios of corn starch and dextrose significantly altered IEAAs losses, thereby affecting the accuracy of the SID and feed formulation. Nevertheless, the underlying reason of how the content of dextrose and starch in NFD affects IEAA loss has not been clearly identified.

The major factors affecting IEAAs flow include feed intake, mucin turnover rate, gut health status, digestive enzyme secretion, environmental and bacterial influence^12^, which might be reflected in the changes of digestive physiology status. Taken together, this study suggested that the IEAAs losses evaluated under normal digestive physiology status are the most representative of the actual IEAAs loss, which is also why the chickens fed on corn-soybean meal served as a control group in this study. This preliminary study aims to explore the best proportion of dextrose and corn starch in NFD to simulate the normal intestinal physiology of broilers, which helps to improve the accuracy of IEAAs determination (Tables 1, 2).

**Table 1:** Ingredient composition and amino acids levels of NFD and control diets (%).

Ingredient	D/CS 1.00	D/CS 0.60	D/CS 0.33	D/CS 0.14	Control^1)^
Dextrose	40.00	30.00	20.00	10.00	0.00
Corn starch	40.00	50.00	60.00	70.00	0.00
Zeolite^2^	8.88	8.88	8.88	8.88	0.00
Cellulose^3^	4.00	4.00	4.00	4.00	0.00
Soybean oil	3.00	3.00	3.00	3.00	3.00
Dicalcium phosphate	1.9	1.9	1.9	1.9	1.9
Limestone	1.00	1.00	1.00	1.00	1.00
Sodium chloride	0.30	0.30	0.30	0.30	0.30
Choline chloride (50%)	0.20	0.20	0.20	0.20	0.20
Vitamin premix^4^	0.02	0.02	0.02	0.02	0.02
Trace mineral premix^5^	0.20	0.20	0.20	0.20	0.20
Titanium dioxide	0.50	0.50	0.50	0.50	0.50
Corn (7.5% CP)	0.00	0.00	0.00	0.00	54.43
Soybean meal (46% CP)	0.00	0.00	0.00	0.00	38.05
DL-methionine (98%)	0.00	0.00	0.00	0.00	0.20
L-lysine HCL	0.00	0.00	0.00	0.00	0.20
**Analyzed amino acids values (as-fed basis)**					
Aspartic acid	0.01	0.01	0.01	0.02	2.16
Threonine	0.00	0.01	0.01	0.01	0.71
Serine	0.00	0.00	0.01	0.01	1.10
Glutamate	0.00	0.01	0.01	0.01	3.60
Glycine	0.00	0.00	0.01	0.01	0.86
Alanine	0.01	0.02	0.02	0.02	0.98
Valine	0.01	0.01	0.01	0.02	0.79
Isoleucine	0.00	0.01	0.01	0.01	0.80
Leucine	0.01	0.02	0.02	0.02	1.60
Tyrosine	0.00	0.01	0.01	0.01	0.71
Phenylalanine	0.01	0.01	0.01	0.02	1.13
Histidine	0.00	0.00	0.00	0.00	0.50
Lysine	0.00	0.01	0.01	0.01	1.28
Arginine	0.00	0.01	0.01	0.01	1.38
Proline	0.01	0.01	0.01	0.02	1.21

^1^Nutrient level of the control diet: Metabolizable energy 2.95MC/kg, Crude protein 22.50%, Ca 1%, non-phytate phosphorous 0.45%.

^2^The zeolite (Deheng mineral products Co., Ltd, Shijiazhuang, China) was used as premix carrier in the NFD diets, it contains almost no protein, energy or any other digestible nutrients.

^3^Sodium carboxymethyl cellulose (CAS: 9004–32-4, Sinopharm Chemical Reagent Co., Ltd, Shanghai China).

^4^Premix vitamin provides per kg of diet: Vitamin A, 10,800 IU; Vitamin D3, 2160 IU; Vitamin E, 4.6 mg; Vitamin K3, 1.0 mg; Vitamin B1, 0.4 mg; Vitamin B2, 5 mg; Vitamin B12, 6 mg; folic acid, 0.1 mg; niacin, 7 mg; pantothenic acid, 5 mg.

^5^Trace mineral premix provides per kg of diet: Cu, 6 mg; Zn, 50 mg; Fe, 60 mg; Fe, 0.15 mg; I, 0.35 mg.

**Table 2:** Nucleotide sequence of primers for gene expression analysis.

Target gene	F:forward, R: reverse	Primer sequence (5' → 3')	Accession no.	Size (bp)
*β-actin*	F	TGTTACCAACACCCACACCC	NM_205518	110
	R	TCCTGAGTCAAGCGCCAAAA		
*SGLT-1*	F	CATCTTCCGAGATGCTGTCA	XM_015275173	169
	R	CAGGTATCCGCACATCACAC		
*GLUT-2*	F	CCGCAGAAGGTGATAGAAGC	NM_207178	87
	R	ATTGTCCCTGGAGGTGTT		
*Mucin-2*	F	TCACCCTGCATGGATACTTGCTCA	NM_001318434.1	228
	R	TGTCCATCTGCCTGAATCACAGGT		

*SGLT-1*: Na( +)-glucose cotransporter 1;*GLUT-2*: Glucose transporter type 2.

## Results

### Serum metabolites

It was found that the serum concentrations of albumin and uric acid were significantly decreased in all NFD groups when compared with the control group (Table 3, *P* < 0.001). The concentration of IGF-1 was significantly decreased in group D/CS 1.00, group D/CS 0.60, and group D/CS 0.14 when compared to the control (*P* < 0.05). However, the content of IGF-1was not changed significantly between group D/CS 0.33 and the control. There were no statistical differences in insulin, glucose, glucagon, and TP levels among groups.

**Table 3:** The effects of different NFD on serum biochemical parameters of broiler chickens.

Item	D/CS 1.00	D/CS 0.60	D/CS 0.33	D/CS 0.14	Control	SEM	*P*-value
Glucose (mmol/L)	12.59	13.59	12.19	12.58	13.20	0.930	0.548
Insulin (IU/mL)	5.72	6.45	7.36	6.73	6.98	0.218	0.206
Glucagon (pg/mL)	159.2	151.4	156.4	143.5	133.2	5.862	0.686
IGF-1 (ng/mL)	16.26^c^	16.86^bc^	19.04^ab^	15.47^c^	21.04^a^	1.019	0.008
TP (g/L)	24.10	23.75	23.83	22.47	21.32	0.548	0.466
Albumin (g/L)	9.10^b^	8.42^b^	9.53^b^	8.88^b^	13.10^a^	0.210	< 0.001
Uric acid (μmol/L)	143.7^b^	134.3^b^	154.8^b^	130.2^b^	254.3^a^	7.719	< 0.001

IGF-1: Insulin-like growth factor-1; TP: Total protein.

Values are expressed as the mean and pooled SEM (*n* = 6 per group). Different superscript letters in the same row mean significant differences (*P* < 0.05).

### IEAAs losses

The results of IEAAs losses indicated that Glu was the most abundant AA in IEAAs flow among all NFD groups, followed by Ser (Table 4). Other AAs present in relatively high concentrations were Thr, Asp, Leu, and His, but ranking differed among groups, indicating the D/CS ratio changed the composition of IEAAs flow to a certain extent. Additionally, a higher ratio of D/CS (1.00 and 0.60) increased the endogenous losses of most AA than that of the lower ratio of D/CS (0.33 and 0.14), including Asp, Thr, Ser, Glu, Gly, Ala, Val, Ile, Leu, His, Tyr, Arg, and Pro (*P* < 0.05), suggesting that the NFD with higher dextrose content could increase the IEAAs losses of chickens.

**Table 4:** The basic IEAAs losses (mg/kg DM intake) in ileum of broiler chickens.

AA	D/CS 1.00	D/CS 0.60	D/CS 0.33	D/CS 0.14	SEM	*P-*value
Asp	1106^a^	1134^a^	798.6^b^	761.4^b^	30.333	< 0.001
Thr	1119^a^	1030^a^	768.2^b^	620.0^b^	25.183	< 0.001
Ser	1223^a^	1270^a^	930.7^b^	826.6^b^	33.660	< 0.001
Glu	2158^a^	2064^a^	1447^b^	1309^b^	69.990	< 0.001
Gly	614.3^a^	622.3^a^	437.9^b^	408.6^b^	56.650	< 0.001
Ala	569.4^a^	526.3^a^	307.5^b^	293.9^b^	21.043	< 0.001
Val	636.6^a^	635.1^a^	438.8^b^	436.9^b^	57.146	0.001
Ile	431.2^a^	462.0^a^	333.7^b^	309.0^b^	16.455	0.009
Leu	812.2^a^	861.5^a^	614.6^b^	580.4^b^	26.852	0.002
Tyr	479.0^a^	491.4^a^	342.0^b^	330.1^b^	14.768	0.001
Phe	455.7^ab^	560.7^a^	393.4^b^	357.6^b^	20.619	0.013
His	733.2^a^	745.1^a^	557.3^c^	645.9^b^	14.189	< 0.001
Lys	596.3	580.7	427.8	411.2	49.008	0.060
Arg	558.0^a^	549.3^a^	379.5^b^	323.8^b^	59.439	0.004
Pro	690.0^a^	656.7^a^	459.3^b^	476.2^b^	30.549	0.025

Values are expressed as the mean and pooled SEM (n = 6 per group). Different superscript letters in the same row mean significant differences (*P* < 0.05).

### Intestinal morphology and goblet cells abundance

As shown in Table 5, there were no significant differences in the villus height and crypt depth in the duodenum and jejunum among groups. In the ileum, the villus height had an upward trend in NFD groups when compared with the control (0.05 < *P* < 0.1), but the opposite trend was observed for crypt depth (0.05 < *P* < 0.1). All NFD groups showed a significant rise in the ileal villus height/crypt depth ratio (V/C) when compared to the control (*P* < 0.05). Moreover, the number of goblet cells in jejunal and ileal villi of all NFD groups was significantly increased when compared to the control (*P* < 0.05).

**Table 5:** The effects of NFD on intestinal morphology of broiler chickens.

Item	D/CS 1.00	D/CS 0.60	D/CS 0.33	D/CS 0.14	Control	SEM	*P*-value
**Duodenum**							
Villus height (μm)	1632	1539	1550	1573	1436	36.120	0.550
Crypt depth (μm)	154.3	166.3	158.2	168.7	174.3	3.828	0.497
V/C^1^	10.76	9.40	9.96	9.61	8.48	0.235	0.067
Goblet cells/100 μm^2^	7	9	7	8	8	0.930	0.331
**Jejunum**							
Villus height (μm)	1124	1211	1101	1189	1106	25.040	0.534
Crypt depth (μm)	136.0	160.0	143.8	145.8	161.3	3.712	0.268
V/C	8.50	7.91	7.78	8.34	7.02	0.187	0.152
Goblet cells /100 μm	11^a^	12^a^	10^a^	10^a^	8^b^	0.227	0.001
**Ileum**							
Villus height(μm)	661.0	800.8	647.6	684.6	644.4	19.992	0.097
Crypt depth(μm)	122.6	133.3	125.3	127.7	146.9	2.500	0.077
V/C	5.46^a^	6.06^a^	5.26^a^	5.47^a^	4.47^b^	0.112	0.008
Goblet cells/100 μm	11^a^	12^a^	12^a^	13^a^	9^b^	0.277	0.022

^1^ V/C: The ratio of villus height to crypt depth.

^2^The number of goblet cells were quantified by counting the number of stained goblet cells per 100 μm length of villus, and present as the average number of goblet cells per 8 intestinal villi.

Values are expressed as the mean and pooled SEM (*n* = 6 per group). Different superscript letters in the same row mean significant differences (*P* < 0.05).

### Digestive enzymes

As shown in Table 6, the maltase activity was significantly higher in both D/CS 1.00 and D/CS 0.60 groups compared to the control (*P* < 0.05). The α-amylase activity showed a significant decrease in all NFD groups when compared with the control (*P* < 0.05). However, the activity of lipase and sucrase in group D/CS 1.00 was significantly higher than control group (*P* < 0.05). Group D/CS 0.60 and group D/CS 0.14 showed higher chymotrypsin activity than the control (*P* < 0.05). These results indicated that increasing the D/CS ratio to 1.00 leads to a higher activity of lipase, sucrase, and maltase, but the activity of most digestive enzymes can be similar to the control group by decreasing the D/CS ratio to 0.33.

**Table 6:** The effects of NFD on the ileal digestive enzyme activity of broiler chickens.

Item	D/CS 1.00	D/CS 0.60	D/CS 0.33	D/CS 0.14	Control	SEM	*P*-value
Maltase (U/mg prot)	112.3^a^	105.0^ab^	87.31^abc^	81.01^bc^	77.34^c^	3.885	0.033
Sucrase (U/mg prot)	52.37^a^	23.70^bc^	28.00^b^	6.69^c^	9.98^bc^	2.812	< 0.001
Lipase (U/g prot)	138.4^a^	50.80^b^	43.15^b^	33.64^b^	79.66^b^	6.713	< 0.001
α-amylase (U/mg prot)	4.32^b^	3.45^b^	7.18^b^	7.30^b^	29.35^a^	1.138	< 0.001
Chymotrypsin (U/mg prot)	13.99^b^	45.61^a^	21.30^ab^	47.36^a^	16.98^b^	3.885	0.029

The enzyme activity of maltase and sucrase were detected in the mucous of ileum, and the enzyme activity of lipase, α-amylase, and chymotrypsin were detected in the digesta of ileum. Values are expressed as the mean and pooled SEM (*n* = 6 per group). Different superscript letters in the same row mean significant differences (*P* < 0.05).

### The AID of DM

**Figure 1:**
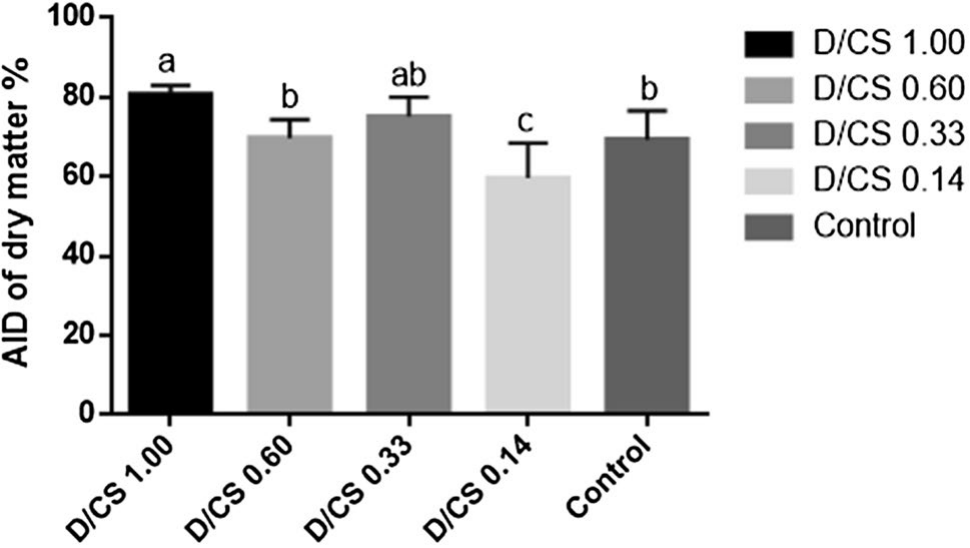
The AID of dry matter in the ileum of broiler chickens (*n* = 6). Labeled means without a common letter are significantly different, *P* < 0.05.

In addition to increasing enzyme activity, group D/CS 1.00 also showed a significant rise in DM digestibility when compared to the control (Fig. 1, *P* < 0.05). There was no significant difference among group D/CS 0.60, group D/CS 0.33, and the control group. However, the DM digestibility in group D/CS 0.14 was remarkably lower than that in the control group (*P* < 0.001), indicating the excess starch is unfavorable for NFD digestion.

### Gene expression

**Figure 2:**
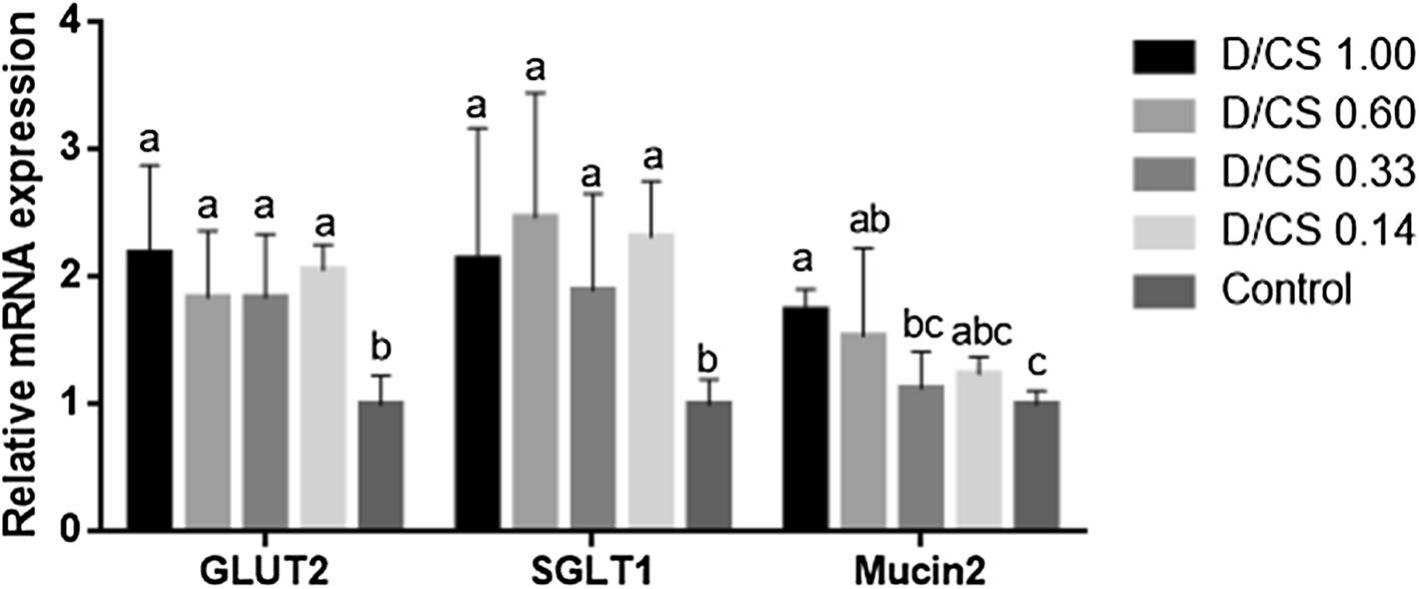
The effects of NFD on the relative gene expression of *GLUT-2*, *SGLT-1* and *Mucin-2* (*n* = 6). Labeled means without a common letter are significantly different, *P* < 0.05.

Mucus secreted by goblet cells is an essential component of endogenous amino acids loss. Therefore, the goblet cell marker gene *Mucin-2* was detected in this study. As shown in Fig. 2, the relative gene expression of *Mucin-2* was significantly higher in the D/CS ratio of 1.00 and 0.60 when compared with the control group (*P* < 0.05). NFD treatments had an upward expression of mucous glucose transporters, with *GLUT-2* and *SGLT-1* levels were significantly higher in all NFD groups than in the control group (*P* < 0.05).

### Microflora analysis of ileal digesta

**Figure 3:**
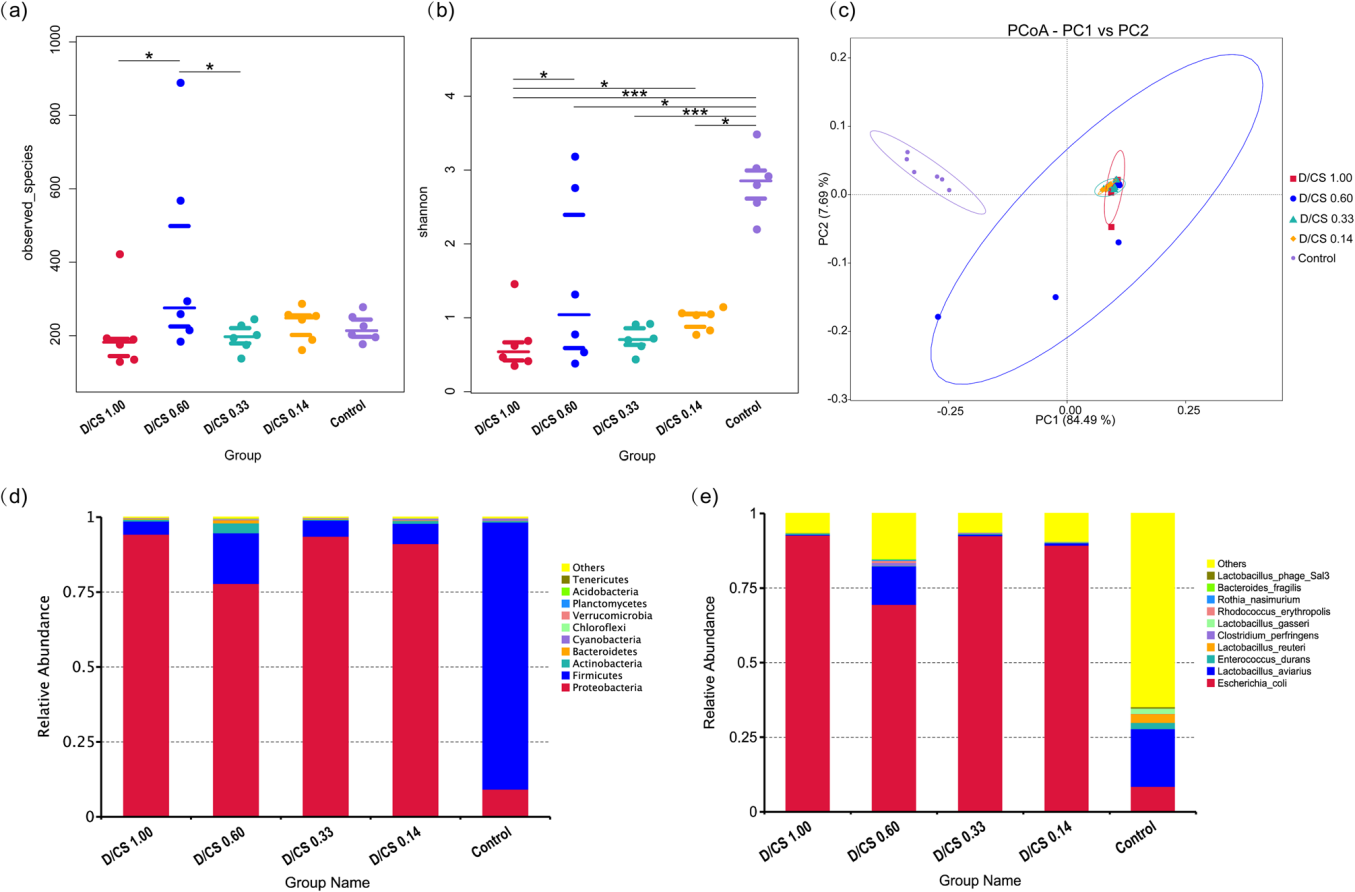
(**a**) Beeswarm of observed species in the ileal digesta among groups, representing the scatter distribution of the total number of species among different groups. (**b**) Beeswarm of Shannon index, reflecting the differences of species diversity and evenness among different groups. (**c**) Principal coordinates analysis (PCoA) of the microbial community. The analysis is generally based on the UniFrac distance, each point in the graph represents a sample, the distance between points represents the degree of difference, and the samples of the same group are represented by the same color. (**d**)The relative abundance of top10 species at phylum level (*n* = 6). (**e**) The relative abundance of top10 species at species level (*n* = 6).

**Figure 4:**
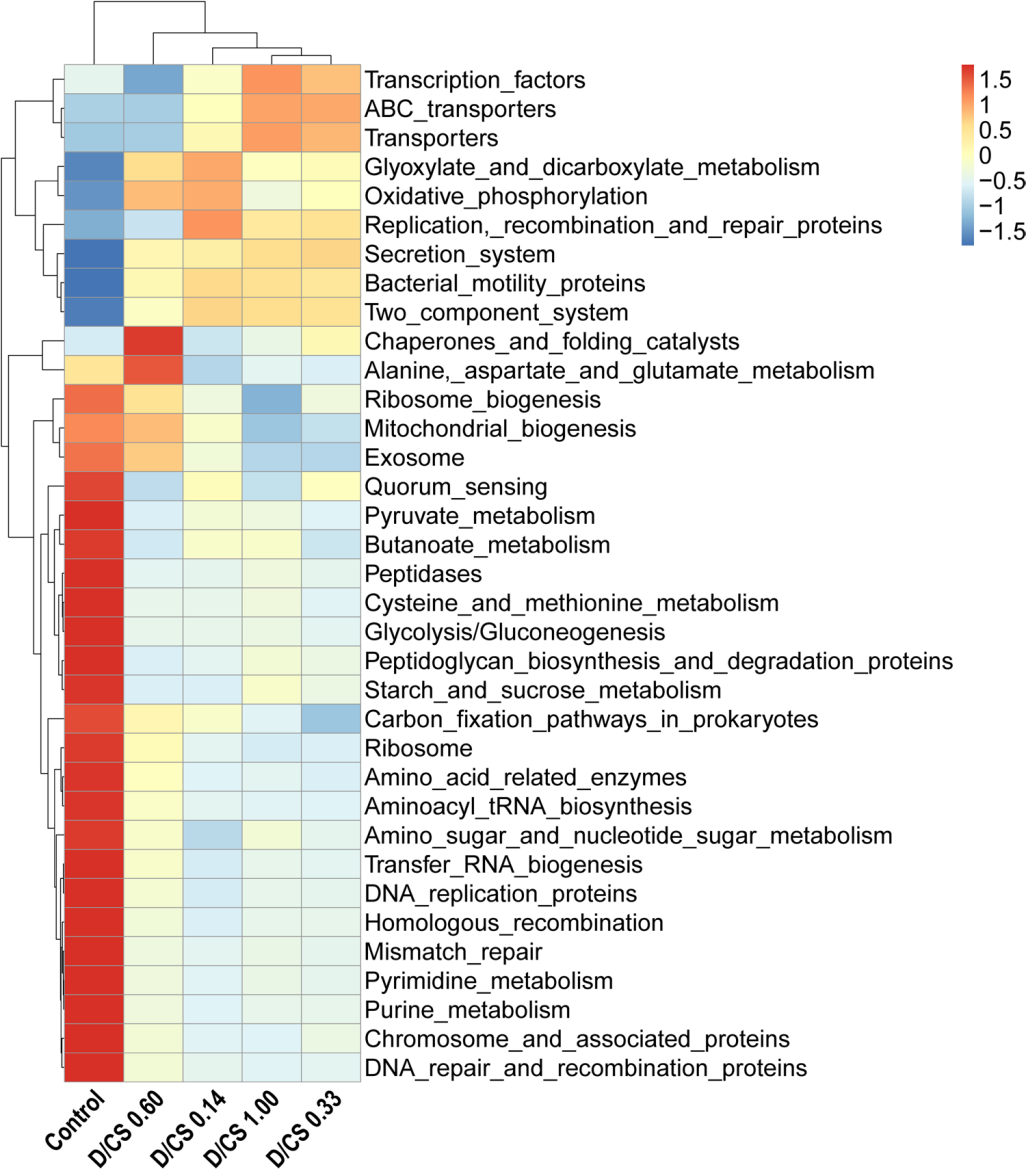
Cluster analysis of the functional relative abundance of Tax4Fun. According to the functional annotation and abundance information of the samples in the database, the top 35 functions and their abundance information in each sample were selected to draw the thermal map (*n* = 6).

According to the results of 16S sequencing based on operational taxonomic units (OTUs) analysis, the microbiota diversity in the ileum of all NFD treated chickens were reduced markedly at different levels (Fig. 3a, b). The principal coordinates analysis (PCoA) of the weighted UniFrac distances resulted in significant segregation among groups (Fig. 3c), confirming the presence of compositional microbiota differences in the ileum of NFD treatments and the control. As shown in Fig. 3d, the preponderant bacteria were *Proteobacteria* (94.29%, 77.87%, 93.63%, 91.18%, respectively) and *Firmicutes* (4.39%, 16.91%, 5.38%, 6.77%, respectively) in the ileum of four NFD groups at the levels of the phylum. However, *Firmicutes* (89.11%) was the preponderant bacteria, which was about tenfold higher than *Proteobacteria* (9.23%) in the control group at the phylum levels. At species level (Fig. 3e), the preponderant bacteria were *Escherichia_coli* (92.76%, 69.57%, 92.52%, 89.38%, respectively) in the ileum of NFD groups. However, the *Lactobacillus_aviarius* (19.32%) and *Escherichia_coli* (8.54%) were the first and second species in ileal microbial communities in the control group, respectively. Considering there were violently disruptive changes of microbiota composition between NFD treatment and the control, it is necessary to analyze the microbial function in this study further. Moreover, the Tax4Fun analysis based on Kyoto Encyclopaedia of Genes and Genomes (KEGG) data predicted differentially expressed functional pathways among groups (Fig. 4).

The four NFD groups harbored very close profiles but differed from those predicted in the control. As shown in Fig. 4, a higher abundance of functions reported for transporters, secretion system, ABC transporters, and two-component system were predicted in NFD groups compared to the control, indicating the microorganism in NFD groups plausibly meets its nutrient requirements for adaption. However, metabolic information related to the DNA repair and recombination protein, the metabolism of amino acids, pyruvate, purine, and pyrimidine appears to be increased in the control group.

## Discussion

There are many methods to determine the IEAAs losses in broiler chickens, such as the regression method, the enzymatically hydrolyzed casein method, the NFD method, and the ^15^N-leucine single-injection method^7,13^. Among these various methods, the NFD method is most widely used for its simplicity, convenience, and low cost. However, one of the fundamental concerns of NFD is its lack of dietary protein, and the animals can be expected to suffer from malnutrition due to the deficiency of essential amino acids.

This study suggested that the IEAAs losses evaluated under normal digestive physiology status represent the actual IEAAs loss, and chickens offered the control diet represent the normal digestive physiological state. Therefore, some serum biochemical parameters were determined in this study to indicate the basic physiological status and metabolic functions of broiler chickens. We found that the albumin and uric acid levels were decreased in all of the NFD groups as compared with the control, indicating clearly that the protein deficiency led to malnutrition of animals in NFD treatments because low albumin and uric acid levels in serum indicating a poor nutritional status^14^ and efficiency of protein retention^15,16^, respectively. Furthermore, serum IGF-1 concentration in these four NFD groups decreased by varying degrees which could reflect some protein restriction because the previous study has shown that serum IGF-1 concentration was reduced in models of severe energy restriction or protein restriction in young growing rats^17^ . However, the levels of glucose, insulin, and glucagon in serum were not influenced by NDF with different ratios of dextrose to starch, suggesting the normal regulation of blood glucose in NDF treatment.

Although changing the ratio of D/CS in NFD did not alleviate the malnutritions of broiler chickens, it did influence the basic IEAAs of broilers in this study. The most abundant amino acids in the ileal endogenous protein of broilers were Glu, Asp, Thr, Pro, Ser, and Gly^18^. Previous research showed that the IEAAs flow depends on the ratio of D/CS in NFD, which increased from 12,779 mg/kg to 17,544 mg/kg of DMI as the proportion of corn starch in NFD decreased from 849.1 to 0 g/kg^10^. Herein, we further narrowed down the ratio of D/CS in the order of 1.00, 0.60, 0.33, and 0.14, and found significant changes when the ratio of D/CS ranged between 0.60 and 0.33, indicating that a higher proportion of dextrose raised ileal IEAAs flow of broiler chickens. These results could be due to the different contributions of sources of endogenous proteins, such as digestive enzyme secretions, mucus, sloughed epithelial cells, and microbial protein.

In the current study, we found that the highest proportion of dextrose (D/CS = 1.00) dramatically increased the activity of lipase, sucrase, and maltase. Excessive digestive enzymes could be reused as either a component of other proteins or recycled after the conservation process^19^, which might be, in part, result in a high level of IEAAs flow. Glucose absorption was mediated by two kinds of transporters, i.e., co-transportation with sodium ions via *SGLT-1* and facilitated diffusion via *GLUT-2*^20^. In this study, we found that NFD rich in glucose and starch significantly increased the gene expression of these two glucose transporters, indicating that the chickens adapted to the absorption of high carbohydrates in NFD. Notably, the digestive enzymatic activity of group D/CS 0.33 resembled closely with the control group, suggesting that the D/CS at 0.33 was potentially appropriate. However, the digestibility of DM significantly dropped when the D/CS ratio was 0.15, which established its unsuitability for the chicken to digest NFD.

In addition to digestive enzymes, mucins also contribute to the IEAAs loss. The mucus secreted from goblet cells constitutes the interface between the gut lumen and the gut epithelium, which is poorly digested in the small intestine^21^. We observed no significant difference in villus height and crypt depth among the treatment groups, but the number of goblet cells was increased on the villus of jejunum and ileum in NFD treatment groups. Moreover, the gene expression of ileal *Mucin-2* was significantly higher in NFD with D/CS at 1.00 at 0.60 than in control. Dietary composition influences the differentiation of intestinal epithelial cells; for instance, diets rich in carbohydrates or AAs lead to different cell differentiation patterns^22^. The nourishment of goblet cells to form mucin depends mainly on the absorbed glutamine and glucose, which is easily provided by the prolamines and starch in grain^23^. Previous in vivo studies indicated that feeding starch increased mucus production in pigs and rats^24,25^. As NFD is rich in starch and dextrose, these results potentially indicate that NFD could promote mucin secretion by increasing the number of goblet cells.

Apart from mucins, microbial protein in the gut also contributes to IEAAs. A high-glucose diet has been linked to gut microbial diversity losses where the proportion of *Bacteroidetes* decreased, while the proportion of *Proteobacteria* was markedly increased^26^. Consistently, in this study, NFD groups with high sugar directly modulate the gut microbiota, characterized by a lower level of *Bacteroidetes* and a higher level of *Proteobacteria*. Since *Proteobacteria* with adherent and invasive properties are considered to be a rich source of lipopolysaccharides (LPS), which has been associated with inflammatory bowel disease and metabolic syndrome^27,28^, we speculate that gut microbiome may be one variable influencing nutrition metabolism function for animals. Therefore, we used Tax4Fun to predict the functional profile of a microbial community and found that NFD increased pathways corresponding to transporters, secretion system, and two-component system, which were closely associated with bacterial adaptation. The adaptation of bacterial species not only relies on the surrounding microenvironment, such as siderophores, exopolysaccharides, protein toxins but also on the two-component system, which allows a pathogen to adapt its gene expression in response to environmental stimuli^29^. Besides, the “secretion systems” facilitate protein toxins transport through the physical barriers that the membranes represent^30^. In this study, the functional prediction of Tax4Fun was consistent with the microbial community changes, indicating that NFD enriched the pathogenic bacteria survival, which represented a considerable health threat to the chicken.

Results of the present study showed that chickens fed with NFD had physiological abnormalities, represented by malnutrition and accumulation of *Proteobacteria* in the gut, which cannot be effectively ameliorated just by adjusting the proportion of starch and dextrose in NFD. To overcome the limitations of NFD, Moughan et al.^31^ proposed an approach that the animal is fed a purified diet containing enzyme-hydrolyzed casein (EHC) as the sole N source to maintain physiologically normal levels of endogenous N flow throughout the intestinal tract. However, the IEAAs losses obtained by this EHC method might be unstable since Ravindran et al.^32^ found that increasing dietary EHC concentrations increased the flow of IEAAs at the terminal ileum of broiler chickens in a dose-dependent manner. Due to the restricted scope of the present study, we did not investigate the effects of supplementing EHC or casein to NFD on the IEAAs of broiler chickens. However, it is worth performing more investigations on combining the advantages of different methods to optimize the detection method of IEAAs in the future.

## Conclusion

Taken together, the present results demonstrated that a higher proportion of dextrose (D/CS = 1 and 0.6) in NFD increases IEAAs by promoting digestive enzymes and mucin secretion. However, the excessive proportion of starch was unsuitable for the chicken to digest NFD (D/CS = 0.14). The broilers in D/CS 0.33 group were closer to the normal digestive physiological state. Thus, the D/CS in NFD at 0.33 might be more appropriate to detect IEAAs of broiler chickens.

## Methods

### Experimental design, animals, and animal care

The animal care and experimental procedures described in this experiment were conducted according to the Animal Welfare Committee guidelines and had the approval of Ethics committee of Animal Science and Technology College of China Agricultural University (No.AW11059102-1, Beijing, China). And the experiments were performed in accordance with the ARRIVE guidelines (https://arriveguidelines.org).

A total of 210,1-d-old broiler chickens were fed the starter diet up to 27 d. At 28-d-old, chickens with similar body weight were allocated to 5 treatment groups with 6 replicate cages (7 chickens per cage) in each group for a 3-day trial period to estimate IEAAs losses. The five treatment groups comprised a control group (corn-soybean meal), and four NFD groups with different ratios of dextrose to corn starch (D/CS), designated as D/CS 1.00, D/CS 0.60, D/CS 0.33, and D/CS 0.14. We found that the starch content in the control diet was 38.76%, and it was challenging to make a pelleted diet with starch content less than 30% in NFD after the pretest. Therefore, the variation of starch content in NFD was based on 40% in this study to make it feasible to prepare the experimental diet.

The content of starch in the feed was measured according to the instruction of a commercial kit for starch content detection (A148-1–1, Nanjing Jiancheng Bioengineering Institute, Nanjing, China). All the diets were pelleted and contained 0.5% titanium dioxide as an indigestible marker for calculating the IEAAs. The reason why 8.88% zeolite was added to NFD is that the zeolite effectively reduces the viscosity of feed during granulation and prevents molten glucose and starch from sticking together and blocking the granulator. In addition, zeolite does not contain nutrients such as protein, which can meet the experimental requirements.

The dextrose and corn starch were purchased from Qinhuangdao Lihua starch Co., Ltd (Qinhuangdao, China), satisfying the corresponding China National Standard GB/T 8885 and GB/T 20,880, respectively. The ingredient composition and AA levels of NFD and control diets are shown in Table 1.

All chickens were raised in conventional cages, and each group was composed of 6 cages, with 7 chickens in each cage (0.7 m^2^/per cage). The brooding temperature was maintained at 33–35 °C from 1 to 2d, and then the temperature dropped by one degree every two days until 21 °C. The relative humidity was maintained at 65–70% from 1 to 7d, 50–65% from 8 to 31d. The lighting schedule consisted of 24 h from 1 to 2d, 23 h for 3d, 22 h for 4d, 21 h for 5d, and 20 h from 6 to 31d. All birds had free access to feed and water. No animal deaths occurred during the 3-day trial period.

### Sample collection

On day 31, one chicken from each replicate was randomly selected to collect blood from the brachial vein and subsequently centrifuged at 1500 × g for 10 min to harvest serum. Afterward, these chickens were euthanatized by injecting pentobarbital sodium (50 mg/kg body weight). The intestine was immediately removed and demarcated by the end of the duodenal loop, Meckel’s diverticulum, and the ileocecal junction. Approximately 1 cm intestinal segment was excised from the middle portion of the duodenum, jejunum, and ileum and then carefully collected in carnoy fixative (G2312, Solarbio, Japan) for Alcian blue-periodic acid-schiff stain (AB-PAS). Another 1 cm ileum segment was collected after washing with ice-cold PBS, snap-frozen in liquid nitrogen, and stored at -80℃ for subsequent analysis of gene expression. The ileal digesta was gently squeezed from the distal ileum and homogenized using a spatula before being collected in two bacteria-free tubes, and then snap-freezing in liquid nitrogen and stored at − 80 °C for further analysis of gut microbiota and digestive enzyme activity, respectively. After the digesta was flushed out with ice-cold PBS, the entire ileal mucosa was scraped off using sterilized microscope slides, collected in tubes, nap-freezing in liquid nitrogen, and stored at − 80 °C for the further analysis of disaccharidase activity. The remaining six chickens in each cage were euthanatized with pentobarbital sodium (50 mg/kg BW), intestine removed, and the digesta of terminal ileum (lower half of the ileum) was collected and pooled within a cage, immediately stored in − 80 °C overnight and then freeze-dried using a vacuum freeze dryer (FD-2, Biocool, Beijing, China).

### Calculations of IEAAs and AID

After freeze-drying, the diet and digesta were ground and sifted through a 40-mesh sieve to ensure homogeneity. The DM concentration of feed and digesta samples was analyzed based on the methods illustrated in AOAC International^33^. The feed and digesta samples were hydrolyzed using 6 mol/L HCl at 105 °C for 24 h under N atmosphere, and then the AA concentration was measured by Amino Acid Analyzer (A-300, Membrapure, Germany). TiO_2_ was determined using the procedure described by Myers et al. ^34^. Briefly, the homogenized ileal digesta samples were ashed, and then digested using sulphuric acid (7.4 M) and subjected to react with hydrogen peroxide, and the absorbance was measured at 410 nm using a spectrophotometer (Ultrospec 2100 pro, Amersham Biosciences, USA).

The IEAAs were calculated as milligrams of amino acid flow per 1 kg of DM intake using the Eq. (1) by Moughan et al.^35^ The apparent digestibility of DM was calculated using Eq. (2). All the data was expressed on DM basis for calculations.

1 $${\text{IEAAs flow }}\left( {{\text{mg}}/{\text{kg of DM intake}}} \right) = \left( {\frac{{{\text{TiO}}_{{2 {\text{diet}}}} {\text{ \% }}}}{{{\text{TiO}}_{{\text{2 ileal digesta}}} {\text{ \% }}}}} \right) \times AA_{{\text{ileal digesta}}} \left( {{\text{mg}}/{\text{kg of DM}}} \right)$$

2 $${\text{AID of DM }}\left( {\text{\% }} \right) = \left[ {1 - \left( {\frac{{{\text{TiO}}_{{2{\text{ diet}} }} {\text{\% }}}}{{TiO_{{\text{2 ileal digesta}}} {\text{\% }}}}} \right) \times \left( {\frac{{{\text{DM}}_{{\text{ileal digesta}}} \% }}{{{\text{DM}}_{{{\text{diet}}}} \% }}} \right)} \right] \times 100\%$$

where: AA _ileal digesta_ represented the AA concentration in terminal ileal digesta (mg/kg of DM); TiO_2 diet_ and TiO_2 ileal digesta_ represented the TiO_2_ concentrations (%) in the diets and terminal ileal digesta, respectively; DM _diet_ and DM _ileal digesta_ represented the DM concentrations (%) in the diets and terminal ileal digesta, respectively.

### Serum metabolites

The glucose, total protein (TP), albumin, and uric acid levels were determined by an automated biochemical analyzer (TBA-120FR, TOSHIBA, Japan). The insulin-like growth factor-1(IGF-1) was detected by IGF-1 600 ELISA kit (DRG, ELA-4140, Germany). The glucagon (GLUN) and insulin (INS) were detected by automatic radioimmunocounter (XH-6080, Xi'an Nuclear instrument Factory, China).

### Intestinal morphology

The tissue sections and AB-PAS stain of duodenum, jejunum, and ileum of broiler chickens were prepared by Servicebio Co., Ltd (Beijing, China). The intestinal morphology was measured based on eight representative complete villi in the same AB-PAS stained slide. Mucosal villus height was defined as the length from the tip of the villus to the crypt opening, and the associated crypt depth was determined from the crypt opening to the crypt base. The number of goblet cells was quantified by counting the number of stained goblet cells per 100 um length of villus and present as the average number of goblet cells per 10 villus.

### Digestive enzymes

The mucosal tissue (0.2 g) was homogenized (4000 rpm, 10 min) with an Ultra-Turrax homogenizer (JIUPIN-92, JIUPIN, WuXi, China) in 6 volumes of saline (4 °C) to collect the homogenate. According to the instructions, the activities of sucrase and maltase were measured (*n* = 6) using commercial assay kits (A082-21, A082-31, Nanjing Jiancheng Bioengineering Institute, Nanjing, China). Furthermore, the data were collected by optical density (all 505 nm) measurement on a microplate reader (SpectraMax i3x, Molecular Devices, LLC, USA).

The intestinal digesta and mucosal samples were immediately snap-frozen using liquid nitrogen. The intestinal digesta (0.2 g) was homogenized (3500 rpm, 10 min) with an Ultra-Turrax homogenizer (JIUPIN-92, JIUPIN, WuXi, China) in 9 volumes of saline (4 °C) to collect the homogenate. And then, the amylase, chymotrypsin, lipase activity was determined according to the instruction of the kit (C016-1–1, A080-3–1, A054-2–1, Nanjing Jiancheng Bioengineering Institute, Nanjing, China). And the data were collected using a spectrophotometer (Ultrospec 2100 pro, Amersham Biosciences, USA).

### Microflora analysis of ileal digesta

DNA extraction was performed using a QIAampTM Fast DNA Stool Mini Kit (Qiagene, No. 51604). High-throughput sequencing of 16S rDNA gene amplicons was performed by Novogene Biotech Co., Ltd. (Beijing, China) using a NovaSeq PE250 platform (Novogene Biotech Co., Ltd, Beijing, China). The high-quality sequences were clustered into operational taxonomic units (OTUs) at a 97% similarity level, and a total of 1808 OTUs were obtained. And then, the OTUs sequences were annotated with Silva132 database. According to the species annotation, the *α* diversity and *β* diversity were further calculated, and the differences between groups were compared to reveal the different characteristics of microbial community structure under different treatments.

### Gene expression

Total RNA was isolated from ileum using RNA Easy Fast Tissue/Cell Kit (DP451, Tiangen Biotech Co., Ltd, Beijing, China), and the concentration and purity of each sample were determined at 260/280 nm. After that, 1ug of total RNA was reversed into the first-strand cDNA using a kit (RR047A, Takara, Kyoto, Japan). Real-time PCR of mRNA was conducted using the ABI 7500 Fluorescent Quantitative PCR system (Applied Biosystems, Bedford, MA). Each RT reaction was carried out by an SYBR Premix ExTaq kit (RR420A, Takara, Kyoto, Japan). The gene-specific primers were commercially manufactured (Table 2; Sangon Biotech, Shanghai, China), and *β*-actin was chosen as the house-keeping gene. The relative gene expression levels were calculated by the ${2}^{-\Delta \Delta Ct}$ method^36^. In addition, the protocol of melting curve analysis was set as follows: 95 °C for 30 s; 40 cycles of 95 °C for 5 s and 60 °C for 34 s; 15 s for 95 °C, 1 min for 60 °C and 15 s for 95 °C.

### Statistical analysis

The data were analyzed by SPSS, version 20.0 (SPSS, IBM, Chicago, IL, USA). Data distribution was checked by Shapiro–Wilk test. Normally distributed data were analyzed by one-way ANOVA for comparisons among groups and then followed by the Dunnett’s post hoc test. Values are expressed as the mean and pooled SEM (*n* = 6 per group). *P* values less than 0.05 were considered statistically significant, and P values less than 0.01 indicates extremely significant differences.
